# Non-invasive monitoring of *Aspergillus* infections in chronic lung disease patients: a combined serology and HRCT imaging approach

**DOI:** 10.3389/fcimb.2025.1494522

**Published:** 2025-06-27

**Authors:** Rong Zhang, Lei Yang, Hong Fang, Qian Xie, Huan Tang, Lin Chen

**Affiliations:** ^1^ Department of Pulmonary and Critical Care Medicine, Sichuan Provincial People’s Hospital, School of Medicine, University of Electronic Science and Technology of China, Chengdu, China; ^2^ Department of Nephrology and Institute of Nephrology, Sichuan Provincial People’s Hospital, School of Medicine, University of Electronic Science and Technology of China, Sichuan Clinical Research Centre for Kidney Diseases, Chengdu, China; ^3^ Enyang District People’s Hospital of Bazhong, Bazhong, China

**Keywords:** *Aspergillus* infection, chronic lung disease, non-invasive diagnosis, serology, HRCT imaging

## Abstract

**Background:**

Diagnosing *Aspergillus* infections in patients with chronic pulmonary diseases is challenging, particularly in settings where invasive diagnostic tools are limited. This study explores a non-invasive diagnostic approach, combining serological tests and high-resolution computed tomography (HRCT) imaging, to identify patients who may need further invasive evaluation for *Aspergillus* infection.

**Methods:**

This retrospective study included patients with chronic pulmonary diseases from regional centers who experienced acute exacerbations that did not respond to antibacterial therapy, had positive sputum cultures for *Aspergillus* species, and lacked typical invasive radiological features on HRCT. Patients were classified based on clinical data, HRCT imaging, and serological markers (IgG, IgM, galactomannan) to distinguish between *Aspergillus* colonization and clinically diagnosed active infection.

**Results:**

Of the 2,731 patients assessed, 209 met the study criteria: 112 were identified with *Aspergillus* colonization, and 97 with clinically diagnosed *Aspergillus* infection. Patients with active infection had significantly higher *Aspergillus* -specific IgG levels (median 185.47 IU/mL vs. 59.96 IU/mL, *p*<0.001) and higher galactomannan indices, especially those with invasive infection (*p*<0.001). HRCT scores were strongly correlated with the risk of infection. The combination of IgG levels and HRCT scores achieved an AUC (area under the curve) of 0.9 for differentiating infection from colonization and 0.74 for distinguishing different types of *Aspergillus* infections.

**Conclusion:**

This study supports the use of a non-invasive diagnostic approach, combining serological testing and HRCT imaging, to identify patients with chronic lung diseases who have positive sputum cultures for *Aspergillus* and are highly suspected of active infection, such as invasive pulmonary aspergillosis and chronic pulmonary aspergillosis, for further invasive diagnostic evaluation. This method is particularly useful in patients who experience frequent acute exacerbations and are unwilling or unable to undergo invasive diagnostic procedures, helping clinicians identify those who really require further definitive evaluation and thereby reducing unnecessary antifungal treatment.

**Clinical trial registration:**

https://www.clinicaltrials.gov, identifier NCT06379568.

## 1 Introduction

Chronic lung diseases, such as Chronic Obstructive Pulmonary Disease (COPD), represent a significant global health burden due to their progressive nature and the complex management required for associated complications. COPD, a leading cause of death worldwide, significantly impacts patient quality of life and survival due to frequent exacerbations (defined as acute worsening of respiratory symptoms such as dyspnea, increased cough, changes in sputum volume or purulence, or fever-and associated complications) (GBD 2019 Diseases and Injuries Collaborators, 2020). The economic burden of COPD is projected to reach $4.3 trillion over the next 30 years, with substantial costs from managing exacerbations, particularly in China and the United States (Chen et al., 2023). Among these complications, *Aspergillus* colonization and infection are particularly challenging, often occurring in patients with pre-existing lung damage or compromised immune defenses. These conditions can further exacerbate respiratory decline and complicate clinical management strategies, especially in settings where advanced diagnostic tools are not readily available.

The prevalence of *Aspergillus*-related complications in patients with chronic lung diseases, including COPD, varies widely, ranging from 1.3% to 3.9%, depending on geographic location and diagnostic criteria (Hammond et al., 2020). Chronic pulmonary aspergillosis (CPA) specifically affects about 1-2% of patients with structural lung abnormalities or a history of pulmonary infections. In more severe cases, particularly among immunocompromised patients, these infections can progress to invasive pulmonary aspergillosis (IPA), which carries a high untreated mortality rate of 50% to 90% (Otu et al., 2023). The management of these infections is both clinically challenging and economically demanding, requiring prolonged antifungal therapy, frequent monitoring, and often resulting in increased hospital readmissions (Imming et al., 2016).

Diagnosing *Aspergillus*-associated respiratory complications in patients with chronic lung diseases is particularly challenging due to non-specific symptoms that can mimic exacerbations of the underlying disease (Kelly et al., 2020). Traditional diagnostic methods, including microbial culture and histopathology, often yield inconclusive results due to several limitations. Microbial culture, although considered a gold standard in identifying fungal pathogens, frequently results in false-negative results, particularly in cases of *Aspergillus* colonization without active infection. The inability to isolate *Aspergillus* species from respiratory samples is common, particularly when the fungal burden is low, or the sample quality is suboptimal. Additionally, histopathological examination can be similarly challenging, as the characteristic fungal elements of *Aspergillus* may not be easily detectable in tissues, particularly in chronic or localized infections, or when the immune response is suboptimal. Both methods require invasive procedures, and in chronic lung disease patients, repeated invasive procedures such as bronchoscopy and bronchoalveolar lavage (BAL) fluid analysis may not always be feasible. This is often due to the patient’s poor compliance, especially in cases where they experience frequent acute exacerbations and are reluctant to undergo repeated bronchoscopy, leading to potential delays in diagnosis or inappropriate treatment.

Given these diagnostic limitations and the constraints associated with invasive procedures, there is an urgent need for non-invasive, cost-effective diagnostic strategies that can help identify patients at high risk for *Aspergillus* infection. Such strategies are particularly crucial in resource-limited settings or for patients who cannot undergo invasive procedures due to frailty or other medical conditions. However, the lack of clear diagnostic evidence often leads to empirical use of antifungal drugs by physicians based on experience, which can result in inappropriate and unnecessary antifungal treatment. Notably, *Aspergillus* spores are ubiquitous in the environment and are inhaled daily, but their detection in respiratory specimens does not always indicate true colonization or infection. In many cases, these findings may reflect transient exposure without fungal growth or host response (Salazar-Hamm et al., 2022). This highlights the need for more accurate diagnostic approaches to distinguish active infection from mere exposure. The goal of our study aimed to establish a feasible preliminary screening standard using non-invasive test, including *Aspergillus*-specific IgG and GM antigen levels combined with HRCT imaging scores. This approach aims to identify patients who are more likely to benefit from further invasive diagnostic evaluation, such as bronchoscopy, while reducing the unnecessary use of antifungal drugs in patients who are unlikely to have active *Aspergillus* infection. By using this screening method, we hope to avoid the overuse of antifungal therapy, which is common when no definitive diagnostic evidence is available, and thereby improve patient management and reduce healthcare costs.

## 2 Methods

### 2.1 Patient inclusion and exclusion criteria

**Figure 1:**
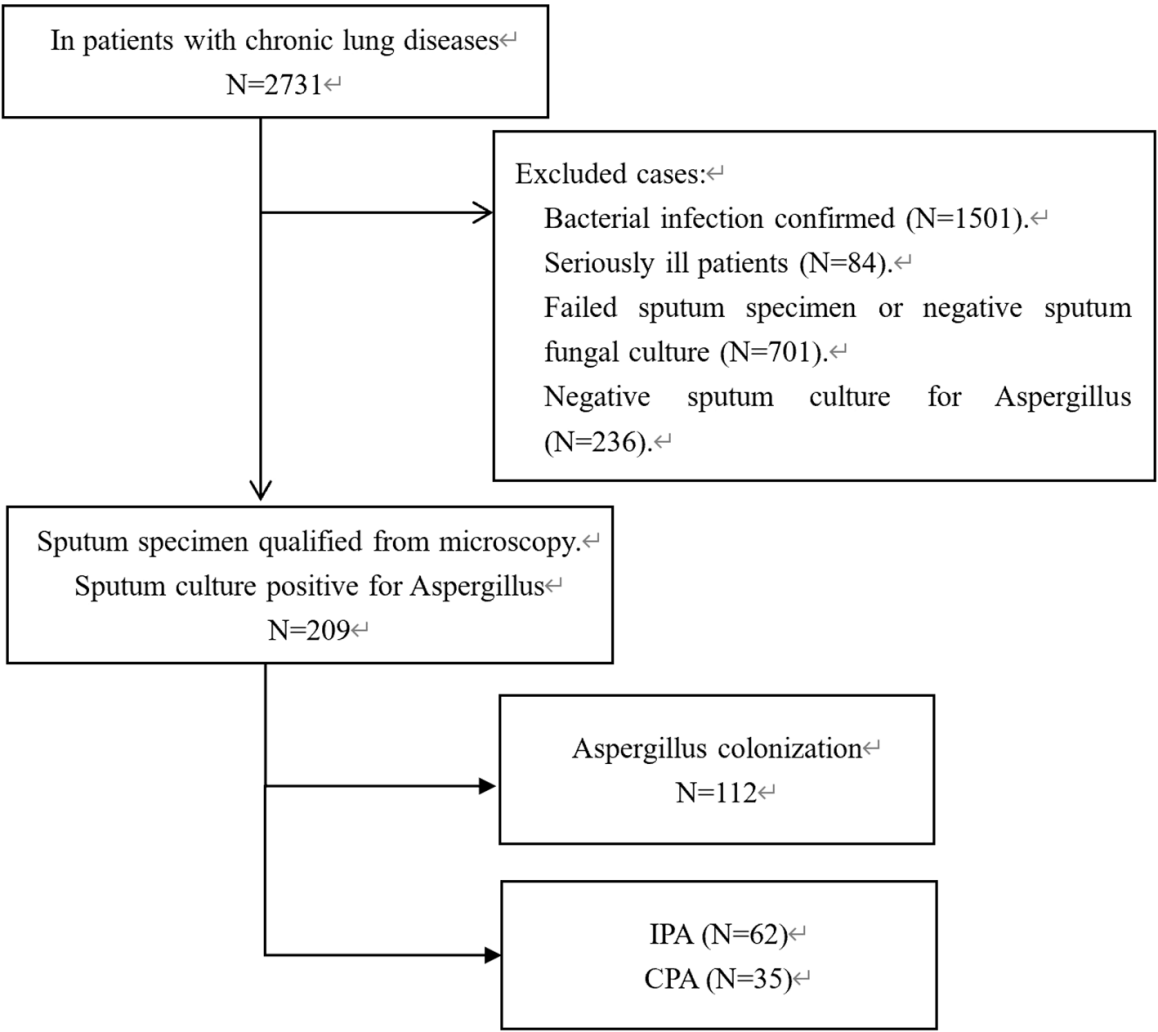
Flow chart for patient inclusion and exclusion. IPA (invasive pulmonary aspergillosis), CPA (chronic pulmonary aspergillosis CPA). Patients who met the exclusion criteria, including those unable to provide adequate sputum samples or those with incomplete clinical data, were excluded from the analysis.

This study recruited 2,731 patients with chronic lung diseases, such as COPD, bronchiectasis, and pulmonary fibrosis, from regional medical centers between January 2019 and December 2022, based on local physicians’ suspicion of *Aspergillus* infection. Clinical specimen collection and initial laboratory testing were performed by participating hospitals according to their routine clinical procedures. Our research team conducted a rigorous quality assessment of all clinical data, excluding unqualified specimens and reports to ensure data quality. The specific exclusion criteria are shown in **Figure 1**. These patients experienced acute exacerbations unresponsive to at least 7 days of standard antibacterial therapy. These samples were collected over four years, including patients from Chengdu and surrounding areas, as well as regions in northern and southern Sichuan.

Despite the suspicion of *Aspergillus* infection, these patients lacked typical HRCT features of IPA or CPA, such as cavitary lesions or nodules, and did not present clinical signs like hemoptysis or chest pain. Due to the unavailability of *Aspergillus*-related serological diagnostic methods at local hospitals, most patients were empirically treated with antifungal therapy, primarily voriconazole. Blood samples were collected from all patients and sent to our center for testing of *Aspergillus*-specific IgG, IgM, and galactomannan (GM).

Patients were excluded if they had severe immunodeficiencies, active malignancies, were undergoing chemotherapy, or had autoimmune disorders requiring immunosuppression. Additional exclusion criteria included patients admitted to intensive care for other conditions, those lacking essential clinical data, those without informed consent, and patients whose symptoms and radiographic findings improved significantly after antibacterial therapy to avoid confounding from mixed infections. Patients unable to provide adequate sputum samples for analysis were also excluded.

The study was approved by a regional ethics committee and registered with ClinicalTrials.gov (ID: NCT06379568).

### 2.2 Patient assessment and data collection methods

#### 2.2.1 General information of patients

Patient assessment involved comprehensive data collection from electronic medical records across all participating hospitals. Clinical data included demographics such as age and gender, physiological indicators like body mass index (BMI), and lifestyle factors such as smoking history. Special attention was given to the history of COPD, including the timing of diagnosis and the frequency of annual exacerbations. Data collection tools incorporated standardized instruments like the COPD Patient Self-Assessment Test (CAT) and the modified Medical Research Council (mMRC) dyspnea questionnaire to evaluate symptoms and functional status. Additionally, treatment details and any complications were meticulously documented to assess the effectiveness of therapeutic interventions and their impact on patient outcomes.

#### 2.2.2 *Aspergillus*-specific serologic tests

Serologic tests played a vital role in diagnosing *Aspergillus* infections, utilizing a chemiluminescence assay for GM detection and a fluorescence immunochromatographic assay for *Aspergillus*-specific IgG and IgM antibodies. Both assays were provided by Dynamiker Biotechnology (Tianjin) Co., with predefined positive thresholds set at 120 AU/mL for IgG and IgM, and 0.6 μg/L for GM. The accuracy and reliability of the serologic data were maintained through rigorous cross-verification with multiple independent data sources, such as electronic health records and laboratory reports. A standardized protocol for data extraction was uniformly applied across all study sites to ensure data consistency. Critical data points were reviewed independently by two researchers, and any discrepancies were resolved through consultation with a third-party expert. The dataset underwent thorough cleaning to address missing values, outliers, and inconsistencies, thereby ensuring a complete and accurate dataset for analysis.

#### 2.2.3 HRCT imaging score

HRCT imaging was a critical component of patient evaluation, providing detailed assessments of lung involvement and the severity of tissue damage. The HRCT scoring system used in this study focused on quantifying these two key parameters consistently. The scoring criteria are summarized in **Table 1** (Bulpa et al., 2007).

**Table 1:** HRCT imaging scoring system for extent of lung involvement and severity of tissue damage.

Scoring Domain	Score	Description
Extent of Lung Involvement	1	No involvement or lesions limited to one lung lobe.
	2	Involvement of two lung lobes.
	3	Involvement of three lung lobes.
	4	Involvement of four lung lobes.
	5	Involvement of all five lung lobes (both sides of the lungs).
Severity of Tissue Damage	1	No observable tissue damage.
	2	Mild emphysema or localized cystic changes with minimal structural distortion.
	3	Moderate structural damage, such as focal bronchiectasis or scattered fibrotic lesions, affecting <40% of the lung.
	4	Extensive bronchiectasis, fibrosis, or consolidation affecting 40–80% of the lung.
	5	Severe tissue damage involving >80% of the lung with structural distortion.
	6	Presence of conglomerate masses, large cavities, or complete architectural destruction (Denning et al., 2016).

This HRCT scoring system was used to semi-quantitatively evaluate the extent of lung involvement and severity of tissue damage in patients with suspected Aspergillus infection. Each HRCT image was independently scored by three radiologists blinded to clinical data, and discrepancies were resolved by consensus. The scores were then averaged for final analysis.

**Figure 2:**
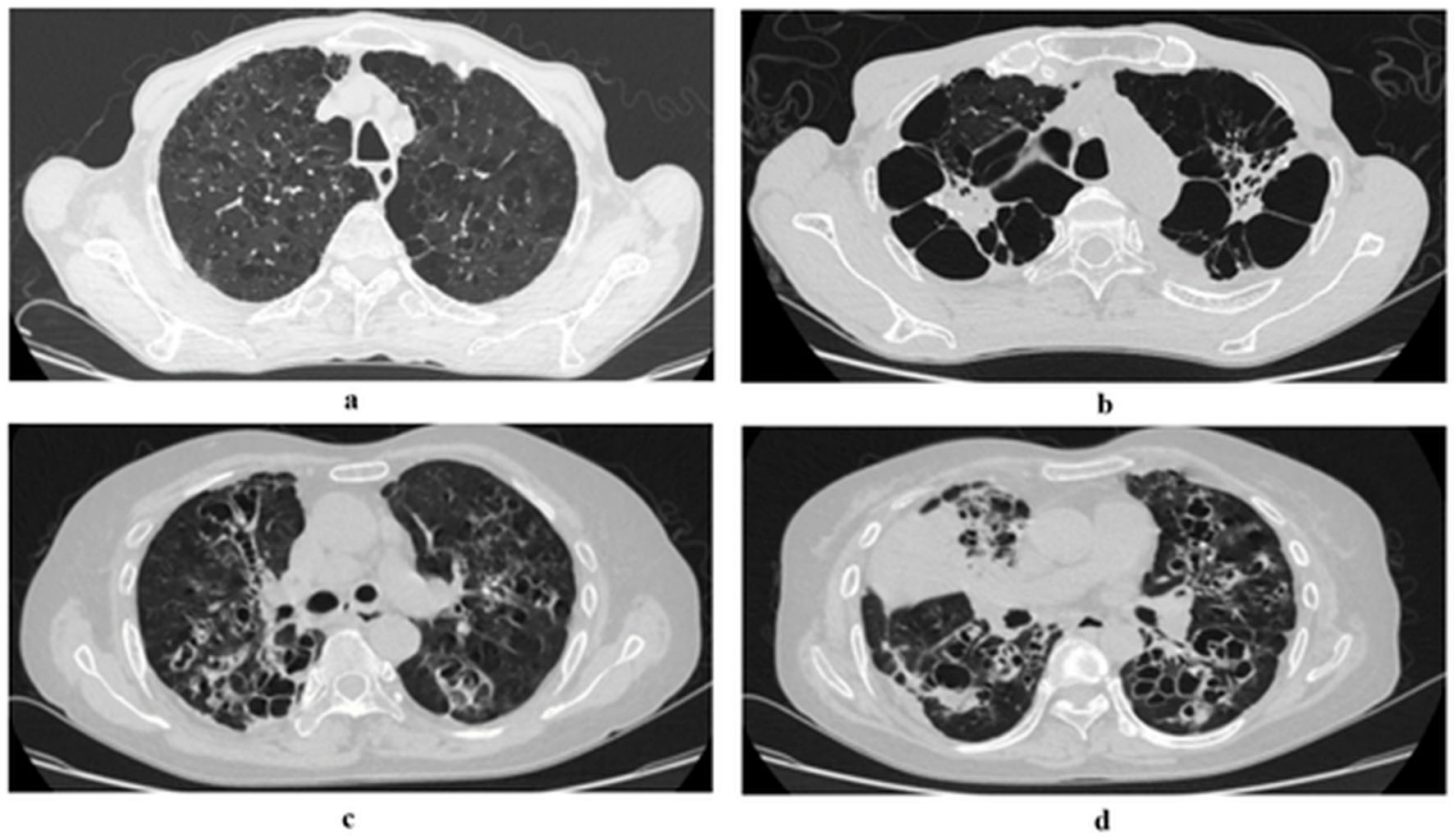
Examples of severity scoring for lung tissue damage. The HRCT scoring system in the provided CT images evaluates lung involvement extent (1–5 scale) and tissue damage severity (1–6 scale). Given that full CT scans are not available for all cases, extent of lung involvement scores are directly presented in the figure. The severity of tissue damage is categorized based on structural destruction and the percentage of lung area affected. **(a)** Mild emphysema and cystic lesions: The image shows mild emphysematous changes with minimal lung involvement. Severity of tissue damage score: 1. **(b)** Severe bronchiectasis with extensive cystic changes: The image demonstrates severe bronchiectasis with multiple cystic structures, significantly affecting both lungs. Severity of tissue damage score: 6. **(c)** Moderate bronchiectasis with widespread involvement: The image indicates moderate bronchiectasis with visible damage covering a substantial portion of the lung but not exceeding 80% of the total lung area. Severity of tissue damage score: 4. **(d)** Severe bronchiectasis with conglomerate masses: The image depicts severe bronchiectasis with complex cystic structures and masses, affecting over 80% of the total lung area. Severity of tissue damage score: 6.

The HRCT images were independently reviewed by three radiologists, each blinded to the patients’ clinical data. The final scores were determined by calculating the mean of the three scores. To assess inter-rater reliability, Cohen’s kappa coefficient was calculated, yielding a kappa value of 0.85, indicating strong agreement. In cases where the difference between any two radiologists’ scores exceeded two points, a fourth senior radiologist was consulted, and a consensus score was established. This approach ensured consistency and reliability in the HRCT scoring process. Examples of the HRCT scoring are shown in **Figure 2**.

An expert panel consisting of three independent respiratory infection specialists conducted an evaluation based on these serological results, along with the patients’ clinical data, medical history, and HRCT findings. Using established diagnostic guidelines, including the Bulpa criteria for IPA (Oda et al., 2014) and the 2016 European Respiratory Society guidelines for CPA (Parkash, 2011), the expert panel classified the patients into *Aspergillus* colonization, clinical diagnosed IPA, or clinical diagnosed CPA. To achieve a more definitive diagnosis, further bronchoscopy had been recommended.

#### 2.2.4 *Aspergillus* identification methods

The identification of *Aspergillus* species was conducted by clinical microbiology laboratories under the jurisdiction of the Sichuan Provincial Bacterial and Fungal Culture Quality Control Center. Isolates were cultured on Sabouraud Dextrose Agar (SDA) plates at 28°C and 35°C, followed by morphological examination to identify isolates to the genus level. Advanced verification was performed using MALDI-TOF MS or Sanger sequencing for species-level identification. Quality control measures ensured the accuracy and reliability of the identification process.

### 2.3 Statistical methods

Statistical analysis was conducted using SPSS software version 25.0. Missing values were addressed by excluding incomplete records from the analysis to ensure data quality and integrity. Continuous variables were expressed as mean ± standard deviation (SD) or median (interquartile range, IQR) depending on the distribution, and compared using the independent samples t-tests or Mann-Whitney U test. Categorical variables were presented as percentages and compared using the chi-square test. Univariate and multivariate logistic regression analyses were used to assess associations between clinical variables and the risk of *Aspergillus* infection. The multivariate model was adjusted for potential confounders. Predictive accuracy was evaluated using receiver operating characteristic (ROC) curves and area under the curve (AUC). AUC values greater than 0.7 were considered indicative of good predictive accuracy. Statistical significance was set at *p* < 0.05.

Although a prospective power analysis was not performed due to the retrospective study design, a *post hoc* power calculation was conducted. Based on the observed differences in *Aspergillus*-specific IgG levels between IPA (n=62) and CPA (n=35) groups, and estimated standard deviations from interquartile ranges (IPA: 56.6 IU/mL; CPA: 71.2 IU/mL), the effect size was approximately 1.16 (Cohen’s d), indicating a large effect. This yielded a power exceeding 95% (α=0.05, two-tailed), confirming that the sample size was sufficient to detect significant differences between groups.

## 3 Results

### 3.1 Basic characteristics of the study population

A total of 2,731 hospitalized patients were initially screened for potential fungal infections. After applying the inclusion and exclusion criteria, 1,501 patients (54.9%) were excluded due to confirmed bacterial infections, and 937 patients (34.3%) were excluded because of either unsuitable sputum samples or negative cultures. Additionally, 84 critically ill patients (3.1%) were excluded based on severity criteria. Ultimately, 209 patients were eligible for the study. Among these, 112 patients (5.1%) were classified into the *Aspergillus* Colonization Group and 97 patients (4.6%) were categorized as having *Aspergillus* infection, which included 62 cases (2.7%) of IPA and 35 cases (1.5%) of CPA. The detailed process of patient selection and exclusion is illustrated in **Figure 1**. To enhance data transparency and reproducibility, key patient-level metadata—including age, gender, clinical group, and laboratory/imaging results (*Aspergillus*-specific IgG, IgM, GM levels, and HRCT scores)—are provided in **Supplementary Table 1**.

### 3.2 Demographic and clinical characteristics of patients with *Aspergillus* colonization and *Aspergillus* infection

The demographic and baseline characteristics of patients with *Aspergillus* colonization and those with *Aspergillus* infection were compared. The average age in both groups ranged between 72 and 74 years, with no significant differences in smoking status, exercise habits, or Activities of Daily Living (ADL) scores (*p*=0.893, 0.45, and 0.05, respectively). The Barthel Index, a measure of independence in daily activities, showed no significant difference between the colonization and infection groups (P=0.061). Gender distribution was predominantly male in both groups, with 70.5% among patients with colonization and 60.8% among those with infection, though this difference was not statistically significant (*p*=0.139). However, within the infection group, a significant difference in gender distribution was observed between patients with IPA and those with CPA (*p*=0.035). The median BMI was higher in the colonization group compared to the infection group (*p*=0.017), while no significant BMI variation was noted between the CPA and IPA subgroups.

Patients with *Aspergillus* colonization had a higher prevalence of COPD (83.9%) compared to those with *Aspergillus* infection (72.2%, *p*=0.001). In contrast, the prevalence of diabetes was higher among patients with *Aspergillus* infection (18.6% vs. 8.9%, *p*=0.042). No significant differences were observed in the rates of pulmonary heart disease or respiratory failure between the two groups (*p*=0.963 and *p*=0.079, respectively).

### 3.3 Imaging and serologic characteristics

HRCT imaging results showed notable differences between the *Aspergillus* colonization and infection groups. Typical lung changes, such as air trapping and nodular cystic changes, were graded on a scale from 1 to 6. Patients with *Aspergillus* infection, particularly those with a grade of 3 or higher, exhibited significantly more severe lung injuries compared to those with colonization (*p* < 0.001). Furthermore, within the infection group, patients with CPA demonstrated more severe lung injuries than those with IPA, as evidenced by higher total imaging scores.

Serologic analysis revealed that *Aspergillus*-specific IgG levels were markedly higher in the infection group compared to the colonization group, suggesting its potential as a diagnostic marker for distinguishing infection from colonization. Notably, patients with CPA had significantly elevated IgG levels compared to those with IPA (224.44 vs. 150.07, *p* < 0.001). IgM levels did not differ significantly between the IPA and CPA subgroups (*p* =0.119). The GM assay results showed higher values in patients with IPA compared to those with CPA and colonization, with significant differences between IPA and CPA (*p* < 0.001) but no significant difference between CPA and colonization.

### 3.4 Longtime treatment patterns and outcomes

Patients with *Aspergillus* infection were more likely to require home oxygen therapy and had longer durations of therapy compared to those with colonization (*p*=0.017 and *p*=0.02, respectively). Patients with infection also used higher doses and longer durations of inhaled corticosteroids, particularly those with CPA (*p*=0.022). Systemic steroid usage was more prevalent among patients with *Aspergillus* infection, with significant differences in total dose and high-dose usage when compared to those with colonization (*p*=0.009 and *p* < 0.001, respectively). No significant differences in systemic steroid exposure were observed between the IPA and CPA subgroups (*p*=0.307). Detailed data on treatment patterns are presented in **Table 2**.

**Table 2:** Comparative analysis of clinical characteristics and treatment in *Aspergillus* colonization and infection groups.

Variables	Colonization (n=112)	Infection (n=97)			*p* value*
		IPA+CPA	IPA (62)	CPA (35)	
Demographics					
Age (years)	72.98 ± 8.21	73.22 ± 8.43	72.53 ± 9.09	74.43 ± 7.09	0.839
Gender (Male %)	79/112 (70.5%)	59/97 (60.8%)	33/62	26/35^@^	0.139
BMI (kg/m²)	22.43 (20.62, 24.22)	20.34 (19.60, 23.92)	20.88 (20.82, 22.45)	19.90 (20.06, 23.03)	**0.017**
Smokers (%)	67/112 (59.8%)	53/97 (54.6%)	29/62	24/35^@^	0.45
Regular exercisers (%)	76/112 (67.9%)	53/97 (54.6%)	35/62	18/35	0.05
ADL (Barthel index)	93.21 ± 14.04	90.26 ± 16.19	91.13 ± 16.86	88.71 ± 15.02	0.061
Comorbidities					
Diabetes (%)	10/112 (8.9%)	18/97 (18.6%)	11/62	7/35	**0.042**
Cancer (%)	4/112 (3.6%)	5/97 (5.2%)	3/62	2/35	0.736
Baseline lung disease (numbers of COPD)	94/112 (83.9%)	70/97 (72.2%)	36/62	24/35	**0.001**
Pulmonary heart disease (%)	80/112 (71.4%)	69/97 (71.1%)	43/62	26/35	0.963
Respiratory failure (%)	15/112 (13.4%)	22/97 (22.7%)	15/62	7/35	0.079
Clinical manifestations and signs					
Wheezing (%)	89/112 (79.5%)	81/97 (83.5%)	51/62	30/35	0.455
Difficulty breathing (%)	105/112 (93.8%)	94/97 (96.9%)	60/62	34/35	0.345
Heart rate	90.12 ± 16.46	95.51 ± 19.08	92.44 ± 16.95	100.94 ± 21.57	**0.045**
Oxygen saturation	93.78 ± 4.39	91.37 ± 7.98	92 ± 7.52	90.26 ± 8.74	**0.006**
Imaging Features & Scoring					
EBLI-HRCT (proportion≥grade 3)	22/112 (19.6%)	63/97 (64.9%)	44/62	30/35^@^	**<0.001**
SBLI-HRCT (more than two lobes)	38/112 (33.9%)	74/97 (76.3%)	38/62	25/35	**<0.001**
Total imaging score on HRCT	3.5(5.13, 7.43)	16 (15.3, 18.95)	15.5 (13.15, 17.79)	20 (17.21, 22.9) ^@^	**<0.001**
Serology					
Specific IgG for *Aspergillus*	59.96 (43.39, 75.86)	185.47 (123.88, 297.53)	150.07 (155.05, 231.53)	224.44 (236.81, 332.93) ^@^	**<0.001**
Specific IgM for *Aspergillus*	72.91 (32.19, 141.82)	119.78 (47.24, 119.14)	129.29 (136.01, 206.39)	74.83 (83.66, 163. 95)	**0.005**
Galactomannan assay	0.25 (0.25, 0.37)	0.33 (0.25, 0.67)	0.49 (0.46, 0.71)	0.28 (0.28, 0.43) ^@^	**<0.001**
Treatment					
Home oxygen therapy (%)	34/112 (30.4%)	45/97 (46.4%)	29/62	16/35	**0.017**
Oxygen therapy time (hours): mean	108.04	146.64	156.03	130	**0.02**
Inhalation or not	45/112 (40.2%)	61/97 (62.9%)	34/62	27/35^@^	**0.001**
Use of ICS (dose×time): mean	10.36	21.31	15.21	32.11^@^	0.296
Systemic steroids use in 1 year (%)	99/112 (88.4%)	84/97 (86.6%)	54/62	30/35	0.695
Total dosage of steroids	290 (301.57, 867.93)	440 (634.75, 1010.53)	538 (661.84, 1182.8)	320 (399.27, 892.85)	**0.009**
Systemic steroid dosage >700mg (%)	21/112 (18.8%)	42/97 (43.3%)	29/62	13/35	**<0.001**
Systemic steroid dosage >1000mg	11/112 (9.8%)	31/97 (32.0%)	21/62	10/35	**<0.001**

The table compares two main groups: the Aspergillus Colonization Group and the Aspergillus Infection Group. The infection group is further subdivided into patients with Invasive Pulmonary Aspergillosis (IPA, n=62) and Chronic Pulmonary Aspergillosis (CPA, n=35). The abbreviations used include ADL (Barthel index) for Activities of Daily Living, EBLI-HRCT for Extent of Basic Lung Injury in HRCT grade 3 or higher, SBLI-HRCT for Severity of Basic Lung Injury from HRCT involving more than two lobes, and ICS (dosage × time) for dosage multiplied by time of use of Inhaled Corticosteroids. Data are presented as mean ± standard deviation or median (interquartile range), and diagnostic data as proportions (percentages). P values less than 0.05 are considered statistically significant. * Comparative results between patients with Aspergillus colonization and those with Aspergillus infection. ^@^ Significant difference between IPA and CPA groups, p<0.05. Bolded values indicate statistically significant differences (p < 0.05).

In this study, specific data on antifungal treatment regimens, including the type, dosage, and duration of therapy, were not systematically collected or analyzed. Therefore, the impact of antifungal therapy on patient outcomes, including potential differences between IPA and CPA subgroups, could not be determined. Additionally, the study did not collect data on the overall mortality rate of the study population during the follow-up period. This lack of data limits the ability to assess the direct effects of *Aspergillus* infection on survival and long-term prognosis.

### 3.5 Two-step predictive modeling for differentiating *Aspergillus* infection

To enhance the accuracy of clinical diagnosing *Aspergillus* infections and distinguish between colonization and infection, as well as to differentiate between IPA and CPA, a two-step predictive modeling approach was applied.

First step: differentiating infection from colonization

**Figure 3:**
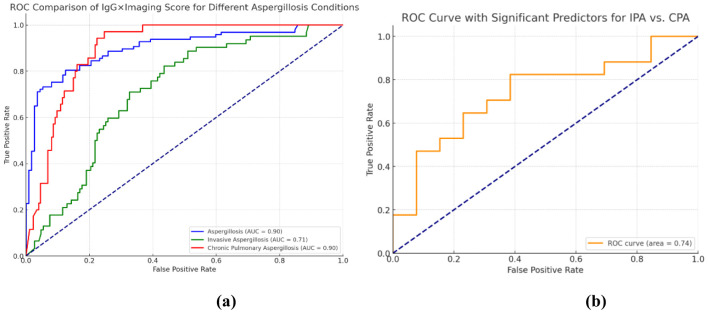
Predictive model for Aspergillus infection and sequential ROC analysis for further differentiation of IPA/CPA subtypes. **(a)** Univariate ROC analysis: The IgG × imaging score is capable of predicting the status of different Aspergillus infections. The blue curve illustrates the capacity of the IgG × imaging score to predict Aspergillosis (AUC = 0.90), while the red curve demonstrates the ability of the score to predict invasive pulmonary Aspergillosis (AUC = 0.71) illustrates the predictive ability of the model to distinguish between chronic pulmonary aspergillosis (AUC = 0.90) and invasive pulmonary aspergillosis (AUC = 0.90). The closer the AUC value is to 1.0, the higher the predictive accuracy. **(b)** demonstrates the differentiation of the predictive modeling between IPA and CPA. The orange curve illustrates the capacity of the model constructed by integrating the three variables, namely IgG, GM test, and imaging score, in differentiating IPA from CPA (AUC = 0.74).

The initial model aimed to distinguish *Aspergillus* infection from colonization among patients with chronic lung diseases. Variables included in the model were inhaled corticosteroid (ICS) usage, systemic corticosteroid doses above 700 mg, HRCT-detected nodular cystic changes, lung damage severity, and serologic markers such as IgG, IgM, and GM levels. The combination of *Aspergillus*-specific IgG levels with HRCT imaging scores demonstrated the highest diagnostic accuracy, with AUC of 0.9 (**Figure 3a**). The detailed statistical analysis of these variables is presented in **Table 3**.

**Table 3:** Diagnostic predictors for different forms of *Aspergillus* infection using AUC scores.

Condition	Predictor	AUC	95% CI
*Aspergillus* infection	IgG×Imaging Score	0.909	0.868 - 0.951
	NCC	0.872	0.770 - 0.885
	EBLI-HRCT	0.819	0.760 - 0.878
	IgG	0.802	0.737 - 0.866
	SBLI-HRCT	0.795	0.732, 0.857
	GM	0.642	0.564, 0.720
	SCS>700mg	0.630	0.551, 0.708
	IgM	0.614	0.535, 0.693
	Inhaled ICS	0.542	0.461, 0.623
IPA	IgG×Imaging Score	0.868	0.810 - 0.927
	NCC	0.835	0.770 - 0.900
	SBLI-HRCT	0.779	0.705 - 0.853
	EBLI-HRCT	0.773	0.694 - 0.852
	IgG	0.741	0.659 - 0.824
	GM	0.706	0.617, 0.796
	IgM	0.666	0.576, 0.756
	SCS >700mg	0.649	0.558, 0.739
CPA	IgG×Imaging Score	0.981	0.962, 1.000
	EBLI-HRCT	0.900	0.846, 0.953
	IgG	0.907	0.859, 0.955
	SBLI-HRCT	0.821	0.737, 0.906
	NCC	0.814	0.732, 0.896
	Inhaled ICS	0.610	0.497, 0.723
	SCS >700mg	0.597	0.483, 0.711
	GM	0.530	0.418, 0.641
	IgM	0.524	0.407, 0.642

IPA, Invasive Pulmonary Aspergillosis; CPA, Chronic Pulmonary Aspergillosis; AUC, Area Under the Curve (a statistical measure of the accuracy of a test or model); CI, Confidence Interval; IgG×Imaging Score, Combined Immunoglobulin G and Imaging Score; NCC, Nodule-Cystic Changes; EBLI-HRCT, Extent of Basic Lung Injury in HRCT; SBLI-HRCT, Severity of Basic Lung Injury from HRCT; GM, Galactomannan; SCS >700mg, Systemic Corticosteroid Therapy exceeding 700mg in 1 year; IgM, Immunoglobulin M; Inhaled ICS, Inhaled Corticosteroids.

Second step: differentiating IPA from CPA

The second phase of the model focused on differentiating between IPA and CPA within the infected group. A logistic regression model incorporating IgG levels, GM levels, and HRCT Imaging Scores was used, achieving an AUC of 0.74 (**Figure 3b**). Among the predictive markers, GM levels showed the highest discriminative power with an AUC of 0.679 (*p*=0.006), followed by IgG with an AUC of 0.708 (p=0.006), and the HRCT Imaging Score with an AUC of 0.65 (*p*=0.018).

### 3.6 The causes of recurrent acute exacerbations in chronic lung disease patients

Further analysis examined patients experiencing recurrent acute exacerbations, defined as two or more exacerbations annually. These patients showed significant differences from those with fewer exacerbations across various clinical and physiological parameters, including Barthel score, independence in daily activities, exercise tolerance, duration of chronic lung disease, CAT and mMRC scores, respiratory rate, heart rate, oxygen saturation, and the duration and dosage of inhaled and systemic steroids. The proportion of patients with recurrent exacerbations was higher among those with *Aspergillus* infection compared to those with *Aspergillus* colonization (61.9% vs. 47.3%), with a statistically significant, though weak, correlation observed in the infection group (R=0.018, *p*=0.006).

## 4 Discussion

This study underscores the diagnostic challenges of clinically identifying *Aspergillus* infections in patients with chronic pulmonary diseases, especially in environments with limited access to advanced diagnostic tools. Among the 2,731 patients initially suspected of having fungal infections, more than half (54.96%) ultimately showed improvement with continued antibacterial treatment, confirming that these cases were bacterial infections rather than fungal. This finding highlights a common issue in primary healthcare settings: the difficulty in differentiating between fungal and bacterial infections due to suboptimal initial antibiotic selection and limited capabilities for pathogen identification. Such diagnostic confusion can lead to unnecessary treatments and delays in appropriate care, emphasizing the critical need for more reliable, non-invasive diagnostic approaches that can be implemented effectively even in resource-limited settings.

Diagnosing *Aspergillus* infections in chronic pulmonary disease patients is further complicated by the limitations of current diagnostic methods. Traditional methods like microbial culture and histopathology are often hindered by long delays, high costs, and technical complexity. GM index levels can vary due to immune status and antifungal treatment, while *Aspergillus*-specific IgG levels have a delayed response. HRCT imaging, though useful, requires skilled professionals and may lack sensitivity in early infection stages. To address these challenges, we propose a simplified scoring system that integrates HRCT imaging, GM antigen levels, and *Aspergillus*-specific IgG levels. This approach provides a more robust evaluation of infection status, particularly in cases with ambiguous clinical presentations.

The combining HRCT and serological testing method was demonstrated by our study to achieve a sensitivity of 80% and specificity of 87% in distinguishing *Aspergillus* colonization from infection. Elevated *Aspergillus*-specific IgG and GM levels are strongly correlated with infection severity, supporting the need for early differentiation of *Aspergillus* colonization and infection (Leav et al., 2000). This approach aligns with previous findings that IgG levels, particularly in non-neutropenic patients, are reliable indicators of *Aspergillus* infection (Sheng et al., 2024). Additionally, IgG was notably higher in patients with CPA, suggesting its utility in identifying infection in chronic lung disease patients. The GM index, while valuable for diagnosing IPA, and the early detection of *Aspergillus*-specific IgM, can provide indications of acute or subacute infections, although with slightly lower diagnostic sensitivity compared to GM.

While HRCT and serologic methods offer significant advantages, they also have limitations. Serological markers such as GM and *Aspergillus*-specific IgG have certain limitations. GM levels can be influenced by the patient’s immune status and antifungal treatment, which may lead to variability in results*. Aspergillus*-specific IgG, on the other hand, has a delayed response, becoming positive only after a period of infection, but it is not affected by treatment (Hage et al., 2019). Moreover, the interpretation of HRCT images requires skilled professionals, and the sensitivity may be insufficient in the early stages of infection. Nevertheless, these non-invasive methods are advantageous over traditional culture and pathology, which, despite being highly specific, are time-consuming, costly, and technically demanding (Alanio and Bretagne, 2017). Cultures take time to yield results, delaying treatment, and histopathology requires specialized knowledge and resources, which may not always be available, especially in certain healthcare settings (Aerts et al., 2024).

The complementary use of HRCT and serology improves diagnostic sensitivity and specify, reducing reliance on time-consuming and costly methods such as culture and histopathology. This combination not only enhances diagnostic accuracy but also optimizes healthcare resource allocation, particularly in settings with limited diagnostic tools. By offering a more personalized diagnosis, this integrated strategy can significantly improve patient outcomes, especially in urgent or resource-constrained situations (Hage et al., 2019; Aerts et al., 2024).

Our analysis also identified several risk factors for *Aspergillus* infection in patients with chronic lung diseases. Consistent with previous findings by Denning et al., we observed that a high cumulative dose of corticosteroids (prednisone >700 mg over three months) significantly increases the risk of IPA (Hage et al., 2019). In our study, patients with *Aspergillus* infection, particularly those with CPA, were more likely to have been on long-term corticosteroid therapy, with significantly higher doses than patients with *Aspergillus* colonization. Other identified risk factors include a lower BMI (20.34 vs. 22.43 kg/m², *p*=0.017), a higher frequency of acute exacerbations (≥2 per year, 61.9% vs. 47.3%), and a greater prevalence of diabetes (18.6% vs. 8.9%). These factors suggest that patients with poorer baseline health or more frequent exacerbations may have an elevated risk of developing *Aspergillus*-related complications, highlighting the importance of targeted prevention and monitoring strategies (Alanio and Bretagne, 2017; Aerts et al., 2024).

Although *Aspergillus* infection rates are relatively low, they remain a significant concern for certain subgroups of patients with chronic lung diseases. Our study observed a higher prevalence of *Aspergillus* colonization in COPD patients (83.9%), with bronchiectasis and pulmonary fibrosis patients showing higher rates of true infection. These findings suggest a need for specific prevention and treatment strategies tailored to different chronic pulmonary disease subgroups. Previous studies have reported varied incidence rates for IPA and CPA among COPD patients, and our findings of 2.27% for IPA and 1.28% for CPA are consistent with these observations (Kosmidis and Denning, 2015; Tiew et al., 2021; Wu et al., 2021).

Despite the relatively low rates of true *Aspergillus* infection, the widespread use of empirical antifungal therapy, especially in regions with limited diagnostic resources, has contributed to the increasing occurrence of antifungal resistance. In our study, among the 209 patients with positive *Aspergillus* cultures, more than half were classified as having colonization and did not require antifungal therapy. This highlights the potential risks of unnecessary antifungal treatment, which can lead to resistance, particularly when the true nature of the infection—whether colonization or active infection—is not clearly established.

A study at the University Medical Center Utrecht found that 16.2% of *A. fumigatus* isolates were resistant to voriconazole in high-risk patients (Fuhren et al., 2015). Similarly, *Aspergillus* species are also facing growing resistance to amphotericin B, further complicating treatment options for these infections (Fakhim et al., 2022). This further emphasizes the need to avoid empirical antifungal treatment, especially in patients with *Aspergillus* colonization, and underscores the importance of confirming the diagnosis before initiating therapy, especially in non-severe patients.

To minimize unnecessary antifungal use, combining HRCT with serological markers like GM and *Aspergillus*-specific IgG offers a more accurate, non-invasive diagnostic approach. However, when active *Aspergillus* infection is suspected, it is essential to conduct more definitive diagnostic procedures, such as bronchoscopy with tissue sampling, to obtain high-quality specimens for further testing, including culture and PCR. This ensures that antifungal therapy is administered only to patients who truly need it, reducing the risk of antifungal resistance while providing appropriate care.

While our study provides valuable insights into the diagnostic approach for *Aspergillus* infection in chronic pulmonary disease patients, several limitations need to be addressed. First, as a multicenter retrospective study, we were unable to completely unify the sample collection and testing processes across all participating hospitals. However, through strict quality control and screening criteria, we ensured the quality of the data included in the analysis. This study design better reflects real-world clinical practice, and the results have greater promotional value for actual clinical applications. Another key limitation is the lack of follow-up data on antifungal treatment outcomes and mortality, which hinders our ability to fully assess the long-term clinical utility of the proposed diagnostic method. A significant factor contributing to this limitation is that many patients in the study were treated empirically with antifungal therapy, and many did not meet the criteria for antifungal treatment based on diagnostic results. As a result, some patients did not receive appropriate treatment, and several others were unable to complete the full course of treatment, making it challenging to assess the effectiveness of antifungal therapy or its impact on mortality.

The primary aim of our study was to establish a simple initial screening method that could help avoid these kinds of unnecessary antifungal treatments. This strategy is particularly important in settings with limited diagnostic resources. However, the next logical step will be to conduct a prospective study focusing on patients with high HRCT scores and significantly elevated serological markers, to identify those who truly need treatment. For these patients, we plan to conduct more comprehensive diagnostic procedures, including bronchoscopy with BALF-GM testing, biopsy, and culture as well as PCR testing, to confirm the diagnosis with high-quality results.

To address these limitations, future research should prioritize several key areas. First, conducting prospective studies with standardized sample collection and testing procedures is essential to further validate the diagnostic accuracy of the proposed method. Such studies would allow for more precise control over variables and provide higher-quality data for analysis. In addition, establishing a more comprehensive follow-up system to collect long-term treatment outcomes and prognosis data would significantly enhance the understanding of the disease’s progression and the effectiveness of interventions. Moreover, investigating the influence of antifungal treatment on serological markers is crucial. This research should aim to develop correction methods to improve the reliability of diagnostic markers, ensuring that treatment does not confound the results. Finally, developing artificial intelligence-assisted HRCT image analysis tools could reduce the reliance on skilled professionals and improve diagnostic sensitivity, making the diagnostic process more accessible and efficient, especially in resource-limited settings.

By incorporating these elements into future research, we can gain a more comprehensive understanding of the clinical utility of this diagnostic strategy and further refine treatment protocols to improve patient outcomes.

## Data Availability

The original contributions presented in the study are included in the article/**Supplementary Material**. Further inquiries can be directed to the corresponding author.

## Supplementary material

Supplementary Figure 1 Proficiency Levels in Aspergillosis-Related Diseases Among Healthcare Professionals from Survey Analysis. Each bar represents the number of healthcare professionals at different knowledge levels (1 through 5) for each disease type, where 1 indicates very low knowledge and 5 indicates very high knowledge.

Supplementary Table 1 Key clinical and laboratory variables (age, gender, imaging, and serologic data) of all 209 patients included in the final analysis. The table supports subgroup classification and ROC model development.
