# Supplementary material for: Non-invasive monitoring of Aspergillus infections in chronic lung disease patients: a combined serology and HRCT imaging approach
Source: Front Cell Infect Microbiol. 2025 Jun 27;15:1494522. doi: 10.3389/fcimb.2025.1494522 (PMC12245828; doi:10.3389/fcimb.2025.1494522)
Supplement: Supplementary Figure 1 — Proficiency Levels in Aspergillosis-Related Diseases Among Healthcare Professionals from Survey Analysis. Each bar represents the number of healthcare professionals at different knowledge levels (1 through 5) for each disease type, where 1 indicates very low knowledge and 5 indicates very high knowledge. [file DataSheet1.docx]

**Supplementary Material: Survey Results and Recommendations on *Aspergillus*-related Diseases Knowledge**

**Survey Results**

A total of 300 surveys were distributed among healthcare workers, with a response rate of 40% (120 complete responses). Participants included general internal medicine physicians, respiratory and critical care specialists, and infectious disease specialists. The survey identified significant gaps in knowledge about high-risk patients, diagnostic techniques, and treatment strategies for *Aspergillus*-related diseases.

**Key Findings:**

- Only 36.67% of respondents used histopathology in diagnosis.
- Awareness of invasive pulmonary aspergillosis (IPA) and chronic pulmonary aspergillosis (CPA) was particularly low among general internal medicine physicians.

**Figures:** Proficiency Levels in Aspergillosis-Related Diseases Among Healthcare Professionals from Survey Analysis.


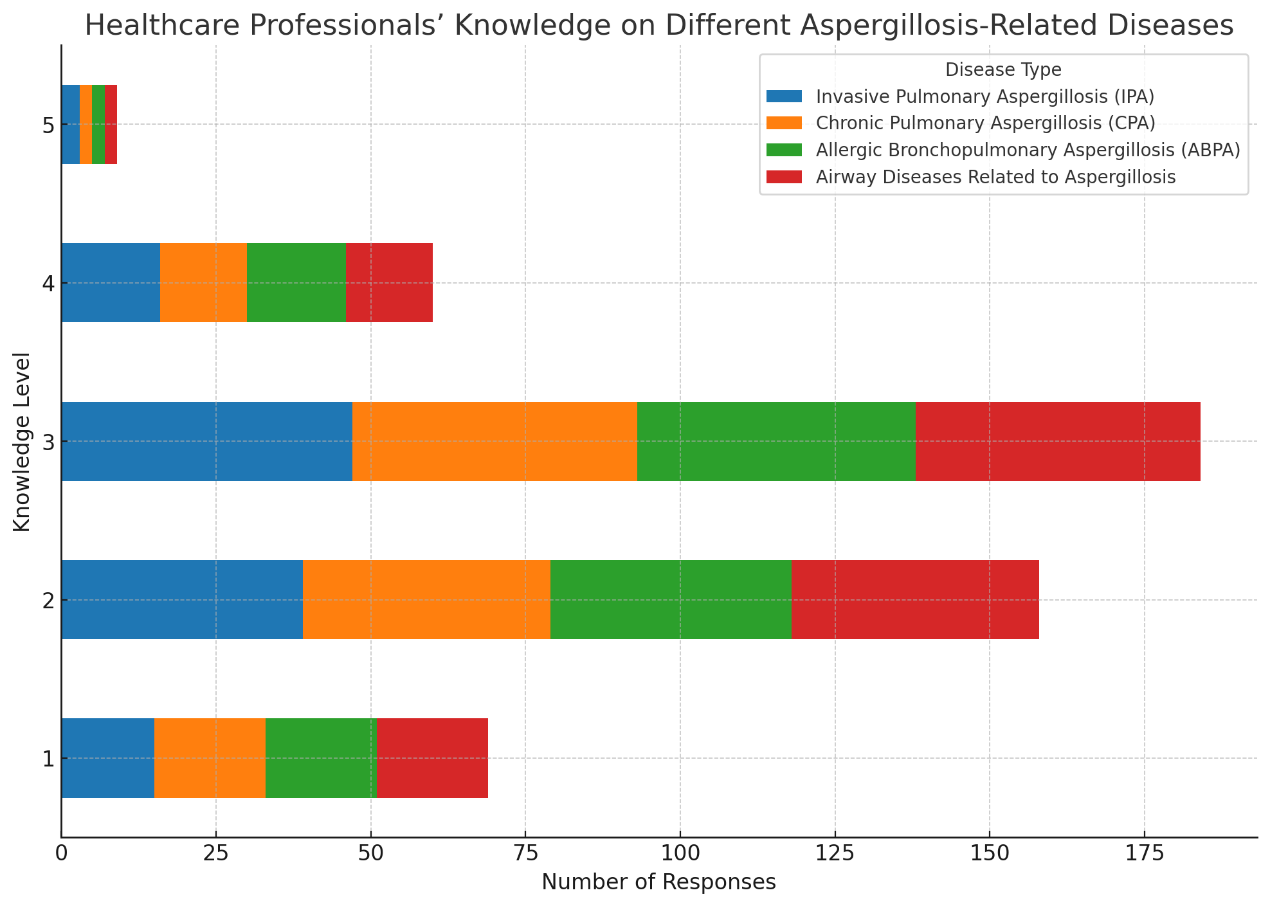


**Figure:** Proficiency Levels in Aspergillosis-Related Diseases Among Healthcare Professionals from Survey Analysis.

**Notes：***Each bar represents the number of healthcare professionals at different knowledge levels (1 through 5) for each disease type, where 1 indicates very low knowledge and 5 indicates very high knowledge.*

**Recommendations:**

1. **Enhanced Educational Programs:** Implement targeted educational programs to improve knowledge about *Aspergillus*-related diseases among healthcare workers, especially in general internal medicine.
2. **Standardized Diagnostic Protocols:** Develop and disseminate standardized diagnostic protocols to ensure accurate and consistent diagnosis of *Aspergillus* infections.
3. **Continuous Professional Development:** Encourage continuous professional development and training on the latest diagnostic and treatment strategies for *Aspergillus*-related diseases.
